# The effect of high pressure on the NMDA receptor: molecular dynamics simulations

**DOI:** 10.1038/s41598-019-47102-x

**Published:** 2019-07-25

**Authors:** Alice Bliznyuk, Yoram Grossman, Yevgeny Moskovitz

**Affiliations:** 1 Israel Naval Medical Institute, Haifa, Israel; 2 Department of Physiology and Cell Biology, Faculty of Health Sciences, and Zlotowski Center for Neuroscience, Ben-Gurion University of the Negev, Beer-Sheva, Israel; 3 Institute of Natural Sciences and Mathematics, Ural Federal University, Yekaterinburg, Russia

**Keywords:** Molecular conformation, Biophysical models, Neurophysiology, Molecular modelling

## Abstract

Professional divers exposed to ambient pressures above 11 bar develop the high pressure neurological syndrome (HPNS), manifesting as central nervous system (CNS) hyperexcitability, motor disturbances, sensory impairment, and cognitive deficits. The glutamate-type N-methyl-D-aspartate receptor (NMDAR) has been implicated in the CNS hyperexcitability of HPNS. NMDARs containing different subunits exhibited varying degrees of increased/decreased current at high pressure. The mechanisms underlying this phenomenon remain unclear. We performed 100 ns molecular dynamics (MD) simulations of the NMDAR structure embedded in a dioleoylphosphatidylcholine (DOPC) lipid bilayer solvated in water at 1 bar, hydrostatic 25 bar, and in helium at 25 bar. MD simulations showed that in contrast to hydrostatic pressure, high pressure helium causes substantial distortion of the DOPC membrane due to its accumulation between the two monolayers: reduction of the Sn-1 and Sn-2 DOPC chains and helium-dependent dehydration of the NMDAR pore. Further analysis of important regions of the NMDAR protein such as pore surface (M2 α-helix), Mg^2+^ binding site, and TMD-M4 α-helix revealed significant effects of helium. In contrast with previous models, these and our earlier results suggest that high pressure helium, not hydrostatic pressure *per se*, alters the receptor tertiary structure via protein-lipid interactions. Helium in divers’ breathing mixtures may partially contribute to HPNS symptoms.

## Introduction

To provide background information regarding the underlying biological mechanisms of high pressure physiology, we feel it necessary to reiterate the main concepts and experimental findings described in our previous studies^1–3^. Military and occupational divers who engage in deep diving reach depths greater than 50 m sea water, and are thus exposed to pressures above 6 atmospheres absolute (ATA) or 6 bar, where 1 bar ≅ 10 m sea water. Professional divers in the oil industry perform underwater construction work at an average depth of 200 m. In 1988, professional divers employed by the Comex diving company in the Mediterranean Sea performed the deepest known working dive at a depth of 534 m (53.7 bar)^4^. The deepest known test dive in a dry pressure chamber, to a depth of 701 m (70.5 bar) was carried out at the Comex facility in France in 1992^5^. To avoid oxygen toxicity and nitrogen narcosis, deep divers use a breathing gas mixture known as trimix, which contains varying percentages of oxygen, nitrogen and helium.

Pressures as high as these present the divers’ lungs, viscera, and particularly the nervous system, with a considerable physiological challenge. Diving deeper than 11 bar may result in the high pressure neurological syndrome (HPNS)^6^, which is characterized by cognitive and motor deficits and reversible central nervous system (CNS) hyperexcitability. As observed in humans and in animal models, susceptibility to HPNS depends on the compression rate and the absolute ambient pressure at the maximal maintained depth. The majority of signs and symptoms in HPNS have their origin in disturbances of CNS synaptic activity^7^. Apart from the symptoms which appear during a dive and are usually reversible, professional divers who engage in repetitive deep sea operations over a period of years may also develop permanent memory and motor impairment^8^.

In recent years, the activity of N-methyl-D-aspartate receptors (NMDARs) has been discovered as one of the main underlying mechanisms of CNS hyperexcitability at hight pressure and the symptoms of HPNS^9–14^. NMDARs are responsible for mediating excitatory synaptic transmission within the CNS^15^. They belong to the family of ionotropic glutamate receptors and have 14 different structural subunits. The GluN1 family is encoded by one gene and may present eight different subunits due to alternative RNA splicing mechanisms^16^: GluN1-1a to -4a and GluN1-1b to -4b subunits. In contrast, the GluN2 family is encoded by four different genes: GluN2A to GluN2D subunits. NMDARs have a heterotetrameric “dimer of dimers” structure^17,18^, the most common combination containing two GluN1 and two GluN2 subunits. The distribution of the NMDAR subtypes depends on age and the brain region within the CNS^18–24^. Each NMDAR subtype has its own unique biophysical and pharmacological properties^25,26^.

Previous electrophysiological studies of rat brain slices in high pressure helium showed a significant increase in the synaptic NMDAR response followed by postsynaptic excitability changes^27,28^ and reduced efficiency of Mg^2+^ blockade^28^. Recent molecular studies conducted in our laboratory^1,2,29^ have revealed that different subunit combinations of the NMDAR exhibit different, sometimes antagonistic, current amplitude change under high pressure helium, whereas receptors containing GluN1-4a or GluN1-4b splice variants were observed to mediate dichotomic current responses^2^.

Our previous experimental molecular approach was limited in revealing the mechanisms underlying the complex high pressure modulation of NMDAR activity. We now propose a new theoretical approach to examine the hypothesis that protein conformational changes, induced directly by high pressure or indirectly via protein-lipid (membrane) interactions, are responsible for the NMDAR hyperexcitability induced by elevated pressure. Using molecular dynamics (MD) simulations^29,30^, Moskovitz & Yang^30^ showed that the dioleoylphosphatidylcholine (DOPC) lipid bilayer responds differently to hydrostatic pressure *per se* and noble gases at elevated pressure. They demonstrated that noble gases have different types of lipid membrane inflation that may produce different responses of the NMDAR to pressure. Recent studies have indicated that NMDAR function is strongly modulated by lipid interactions^31,32^, and that the TMD-M4 α-helix plays a crucial role in NMDAR gating^33^. The Mg^2+^ binding site, containing asparagine (Asn) residues at the N and N + 1 sites, is responsible for voltage-dependent blocking of the NMDAR^34,35^. In the first MD simulation of NMDAR, Mesbahi-Vasey *et al*.^36^ suggested that the same sites bind the Ca^2+^ ion and determine the permeability of the pore. In addition, previous computational studies have suggested that the pore solvation pattern could be a key factor in determining channel conductance^37^, and a meticulous analysis of solvent (water) layout will therefore be employed in the present investigation to evaluate the pore condition. However, the question still remains as to the precise effect of elevated pressure on all of the NMDAR regions. We are of the opinion that the answer may be obtained by using MD simulations.

## Results

### RMSD of the NMDAR

**Figure 1:**
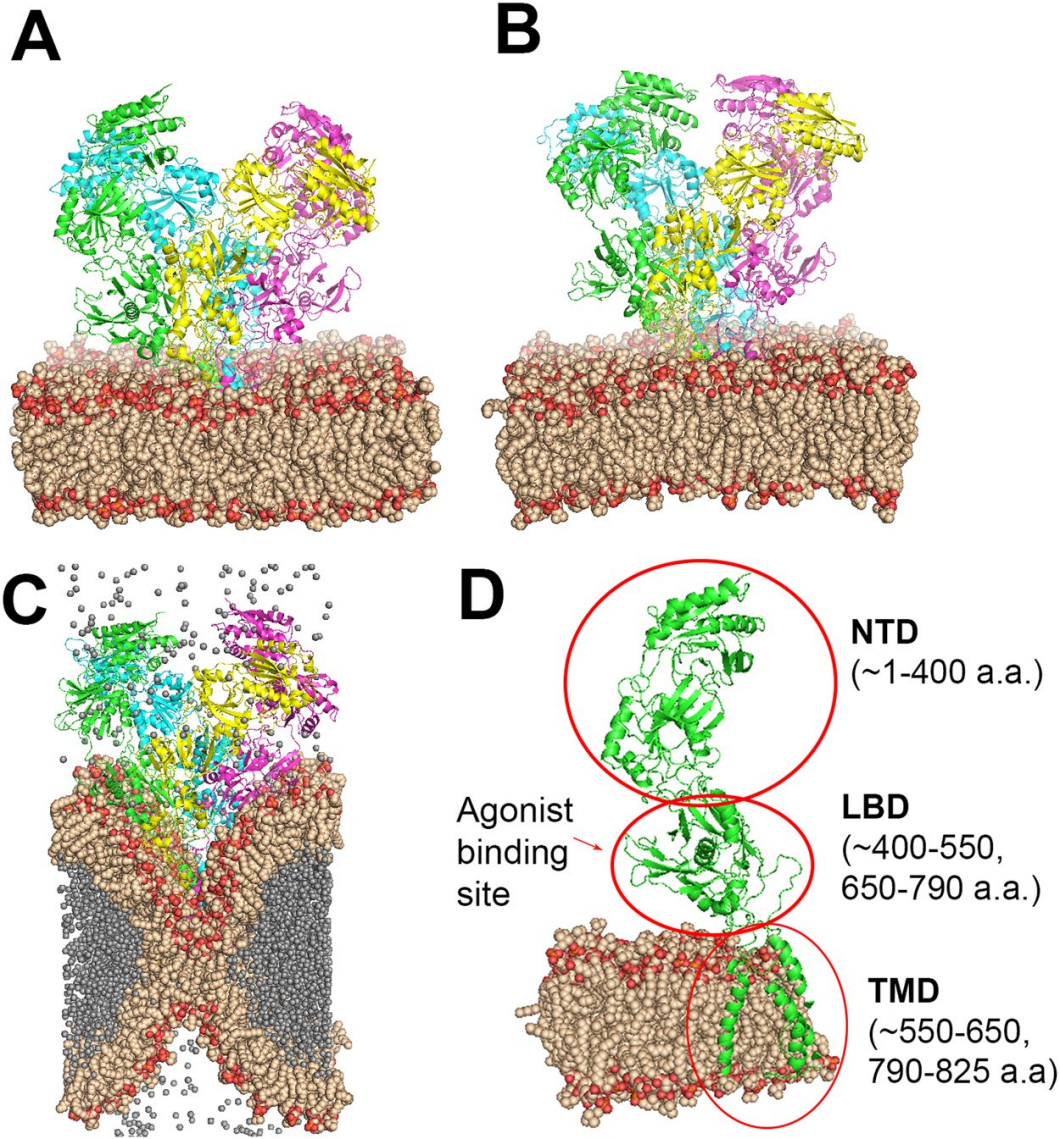
Simulated NMDAR embedded in a DOPC lipid bilayer at different pressures. The NMDAR is shown as cartoon colored by chain (green, magenta, yellow, cyan), DOPC is presented as spheres in wheat and red color (colored by element), helium atoms are presented as spheres in gray. (**A**) 1 bar pressure. (**B**) 25 bar hydrostatic pressure. (**C**) In helium at 25 bar. (**D**) One subunit of the NMDAR in the membrane, important sites indicated by arrows. Numbers in parentheses indicate the a.a. span of the domain (red circle). All GluN subunits share a modular architecture that is composed of four distinct domains: the N-terminal domain (NTD), the ligand-binding domain (LBD) that binds to glycine or d-serine in GluN1 and GluN3, and glutamate in GluN2, the transmembrane domain (TMD) containing the ion channel, and an intracellular C-terminal domain (CTD) (not shown in the figure). The NTD and CTD are the most divergent regions.

**Figure 2:**
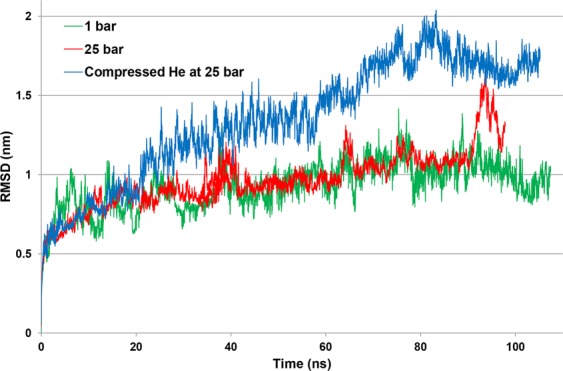
RMSD of the NMDAR during 100 ns of MD simulation. RMSD were calculated as the deviation from the initial NMDAR structure model at 0 ns. Result of the simulation at 1 bar pressure (control) is shown in green, at 25 bar hydrostatic pressure in red, and in helium at 25 bar in blue.

Running the simulation video in slow motion (Fig. 1) showed that helium causes substantial distortion of the membrane. This was also shown for neon during a 200 ns simulation^30^. It is important to note that the percolation time of neon into the DOPC bi-layer was determined to be in the range of 5‒10 ns, and is positively correlated with high pressure. To examine whether helium also causes distortions and changes in the NMDAR, root mean square deviation (RMSD) plots were calculated. RMSD plots indicated that pressurized helium causes protein conformational alteration, whereas hydrostatic pressure *per se* (25 bar) does not induce any change (Fig. 2).

### Cluster analysis

After removing the periodic boundary conditions (PBC), NMDAR cluster RMSD was calculated for each simulation. Calculations were performed for the last 50 ns of the simulation (see Methods). Because of the large variety of protein conformations, resulting in a large number of clusters, we performed the analysis only on the cluster that exhibited the highest probability. Only one frame representing the centroid of that cluster was printed as a PDB file of the simulation box. We visually observed several members of each cluster and found them to be very similar to the centroid.

The data (summarized in Table 1) revealed that the most probable cluster contains representatives from all time frames during the last 50 ns of the simulation. It also revealed that the NMDAR’s most common conformation is very stable under control conditions, with a probability of 0.83 and only a total of 11 smaller clusters. Examining the two high pressure conditions revealed a different situation. Hydrostatic pressure and helium at 25 bar had a total of 31 and 30 clusters, respectively. The largest cluster for hydrostatic pressure had a probability of 0.3, whereas for helium this was only 0.21. Also of interest is the fact that helium had 5 large clusters with a probability of 0.1 or more (probability of clusters 2–5 were: 0.156, 0.124, 0.108 and 0.101 respectively), whereas hydrostatic pressure had only 3 clusters (probability of clusters 2–3 were: 0.180 and 0.135 respectively).

**Table 1:** Summary of the cluster analysis for the simulations between 50 ns and 100 ns.

Gas type	Pressure (bar)	Simulation time (ns)	Total number of frames	No. of clusters	Probability of the biggest cluster	Frames in the biggest cluster	Probability of the other clusters (# of the cluster)	First frame in the biggest cluster (ps)	Middle frame in the biggest cluster (ps)	Last frame in the biggest cluster (ps)
No gas	1	101	14251	11	0.83	11872	0.09 (2)	50232	71092	107000
No gas	25	101	12751	31	0.3	3812	0.18 (2) 0.13 (3) 4-0.08 (4)	62652	84848	99776
He	25	104	13501	30	0.21	2812	0.15 (2) 0.12 (3) 0.10 (4) 0.10 (5)	70988	79612	96168

### Molecular density along the Z axis

**Figure 3:**
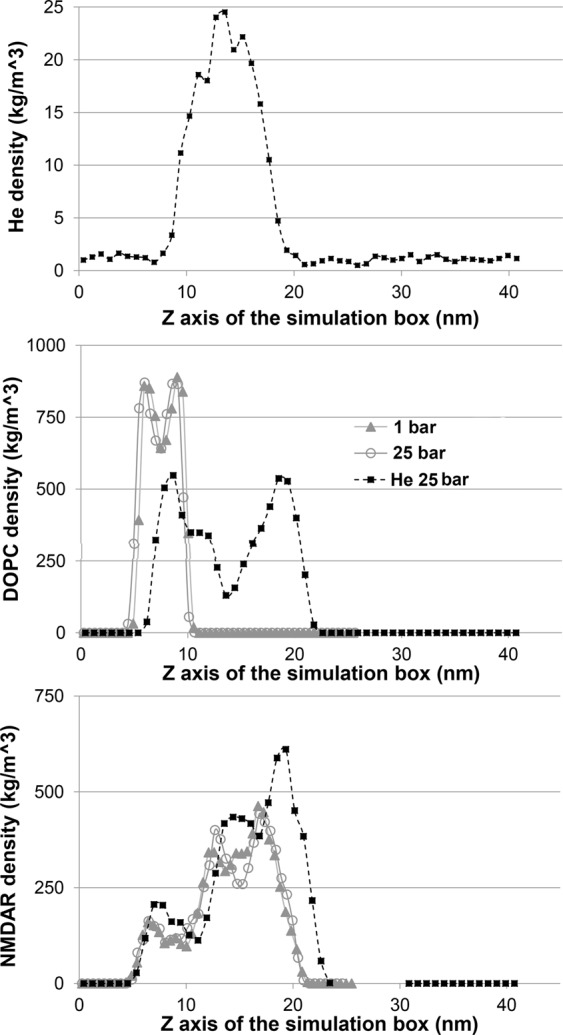
Molecular density calculations along the Z axis. (**A**) Helium density along the Z axis at 25 bar. (**B**) DOPC density at 1 bar (-▲-), at 25 bar hydrostatic pressure (-○-), and in helium at 25 bar (-■-). (**C**) NMDAR density at 1 bar (-▲-), at 25 bar hydrostatic pressure (-○-), and in helium at 25 bar (-■-). See text for detailed explanation.

To gain a better understanding of high pressure helium’s influence on the DOPC and NMDAR, we calculated the density of each component of the simulation box along the Z axis (normal to the membrane surface). As previously reported for neon atoms^30^, the data confirmed that most of the helium atoms cluster between the two bilayers of the DOPC in a hydrophobic pocket that occupies the 10‒20 nm “layer” (Fig. 3A). This result of membrane inflation is reflected in two new DOPC density peaks around the 10 and 20 nm levels (Fig. 3B). In contrast, there is almost no difference between control (1 bar) and 25 bar helium for the density of DOPC that occupies the original “layer” of the membrane, with two close peaks at 5 and 10 nm. The density distribution of NMDAR is unchanged at 25 bar compared with control conditions; however, it increases and is slightly inflated under high pressure helium (Fig. 3C). The dimensions of the simulation box were not preserved under helium; these decreased along the X and Y axes and increased to 40 nm along the Z axis.

### Volume calculation

Volume calculations (Table 2) for DOPC, NMDAR and helium in the simulation box were estimated using a 3V server (see Methods) having a probe radius of 1.4 Å for DOPC/NMDA and 6 Å for the continuous helium phase between two monolayers (the helium-DOPC “bubble”). Whereas there was only a slight variation in the volume of biomolecules at 25 bar (less than 1%, with the exception of helium-saturated DOPC), the considerable helium-DOPC bubble which accumulated within the highly hydrophobic DOPC core at 25 bar increased total DOPC volume (DOPC taken together with the helium-DOPC “bubble”) almost two-fold.

**Table 2:** Molecular volume of biomolecules and helium under different experimental conditions. % change, calculated with respect to 1 bar. Helium DOPC “bubble” refers to helium confined between the two monolayers.

Experiment	DOPC (nm³)	DOPC % change	NMDAR (nm³)	NMDAR % change	Helium DOPC “bubble” (nm³)
1 bar	585.6		489		
25 bar	586.6	0.17	487.8	−0.25	
Helium 25 bar	582	−0.61	489.3	0.07	396.6

### Analysis of helium and water radial distribution functions (RDF)

Given the highly diffusive nature of helium in the polar bulk solvent, water, the distribution of dissolved helium atoms and simple point-charge (SPC) water molecules along NMDAR chains at the immediate protein boundary was studied by applying a series of radial distribution function (RDF) calculations (see Methods). The RDFs’ numerical values stacked as 2D matrices were subsequently averaged over the range 0‒3.4 nm, which is approximately the linear length of a 10 a.a.-long peptide.

**Figure 4:**
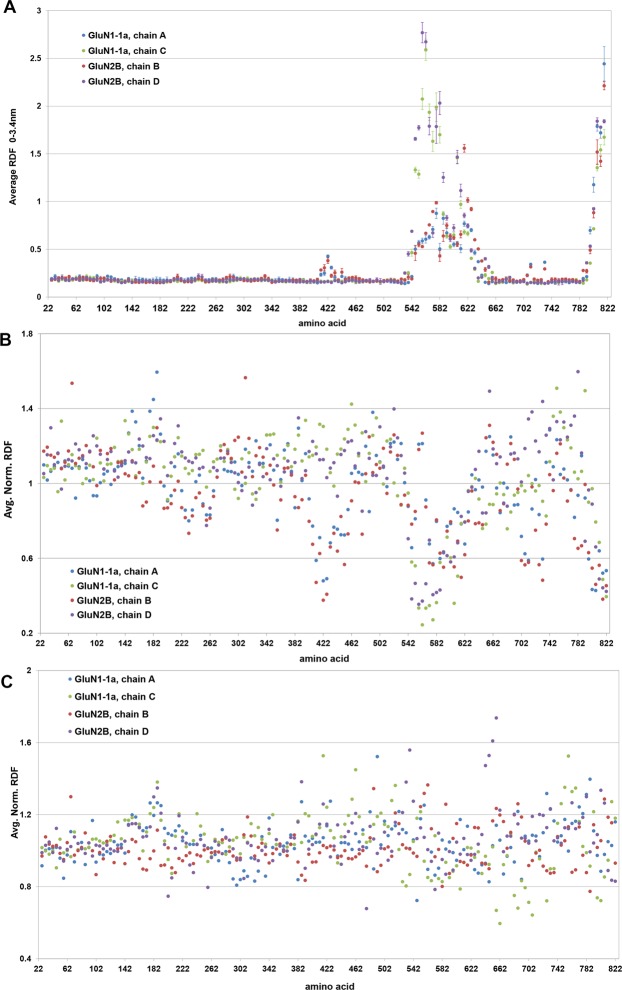
RDF around Cα along A, B, C and D chains of the NMDAR molecule in the range 0‒3.4 nm. (**A**) Average values for helium at 25 bar. (**B**) Values for water RDF in helium at 25 bar normalized to average values for water RDF in control (1 bar). (**C**) Values for water RDF at 25 bar hydrostatic pressure normalized to average values for water RDF in control (1 bar).

We first calculated the layout of helium atoms (Fig. 4A). As demonstrated macroscopically (density plot, Fig. 3A), helium is generally concentrated at the transmembrane domain (TMD). However, the RDF analysis revealed that helium distribution is not uniform among NMDAR segments; C and D TMD chains are highly susceptible to a hydrophobic helium phase (RDF >1.5), and agonist binding domain (ABD) segment 402‒442 is an additional intramolecular helium pool, albeit with a smaller RDF value (0.5). Error bars referencing to the hyperbaric helium layout in the second part of the MD trajectory could provide partial support for large helium bubble stability after its initial penetration into the DOPC gap, because no further large-scale helium fluctuations were observed at t >50 ns along the NMDAR molecule.

As mentioned in the introduction, the pore solvation pattern may be a key factor in determining channel permeability^37^. We therefore analyzed the water RDF along the various chains of the NMDAR molecule. The water solvent layout for control, 25 bar hydrostatic pressure and 25 bar helium is presented in Figs S1–S3, respectively (Supplementary Data). Whereas the water RDF seems undisturbed at 1 bar and its average values distribution narrows at 25 bar, exposure to 25 bar helium results in a more complex solvation pattern. It significantly destabilized water molecules, especially in the vicinity of the GluN2B (B chain); larger error bars in the ABD region (a.a. 382‒482) indicate significant density fluctuation.

To improve the characterization of hydration-dehydration transitions at the protein boundary, we normalized the RDF values at 25 bar hydrostatic pressure and 25 bar helium to the corresponding values under control conditions at 1 bar. The normalized water layout for 25 bar (Fig. 4C) remained mostly linear. The normalized water layout for 25 bar helium (Fig. 4B) showed ‘negative drops’ complementary to the localization of helium RDF peaks (Fig. 4A). This could be attributed to dehydration transitions at the TMD due to diffusive helium accumulated in the bilayer gap.

### DOPC acyl chains order parameter

**Figure 5:**
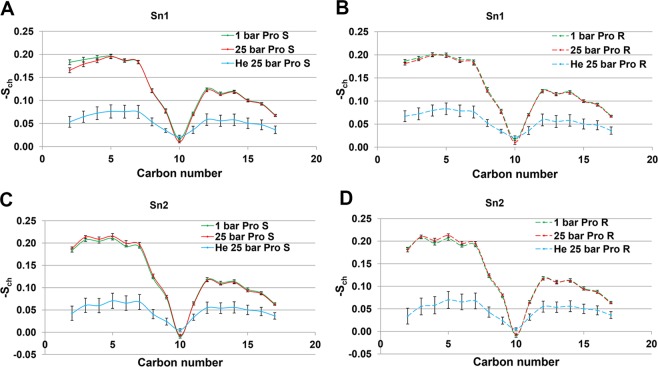
S_ch_ calculated for the Sn-1 and Sn-2 tails of the DOPC simulation. (**A**) Sn1- Pro S. (**B**) Sn1- Pro R. (**C**) Sn2- Pro S. (**D**) Sn2- Pro R.

pro-R and pro-S hydrogen atoms S_ch_ were evaluated separately in each chain, as having nonequivalent order parameters at unsaturated lipids (Fig. 5). As previously reported^38,39^, pro-R values were consistently higher than pro-S values, although this was not statistically significant for the native bilayer distorted only by hydrostatic pressure, with one exception, namely pro-S at C2 at Sn1 under 25 bar. In contrast, bilayer saturation with helium atoms at 25 bar prompted striking separation of the S_ch_ curves compared with the bilayers at 25 bar, followed by a 3‒4 fold reduction in S_ch_ values, whereas C10 double bond exhibited a relatively frozen pattern under all conditions. The C2‒C7 region is also subject to remarkable statistical noise induced by helium, especially at Sn-2, which makes pro-S and pro-R indistinguishable in the presence of dissolved gas along the whole C2‒C17 sequence.

### High pressure effects on pore structure

Simulations at hydrostatic pressure and under high pressure helium showed dissociation of one or more of the agonists. We concluded that the NMDAR was in the closed state. Under control conditions, the NMDAR remained in the open state with four bound agonists in the ABD. Another indicator of pore state is the distance between the Cα atoms of Glu299 in GluN1 (in N-terminal domain) subunits as discovered by Tajima *et al*.^40^. The measurements indicated that in the control simulation the distance was 33.6 Å (open state), at hydrostatic pressure this was 15.19 Å (closed state), and under high pressure helium it was 23.43 Å (closed state).

**Figure 6:**
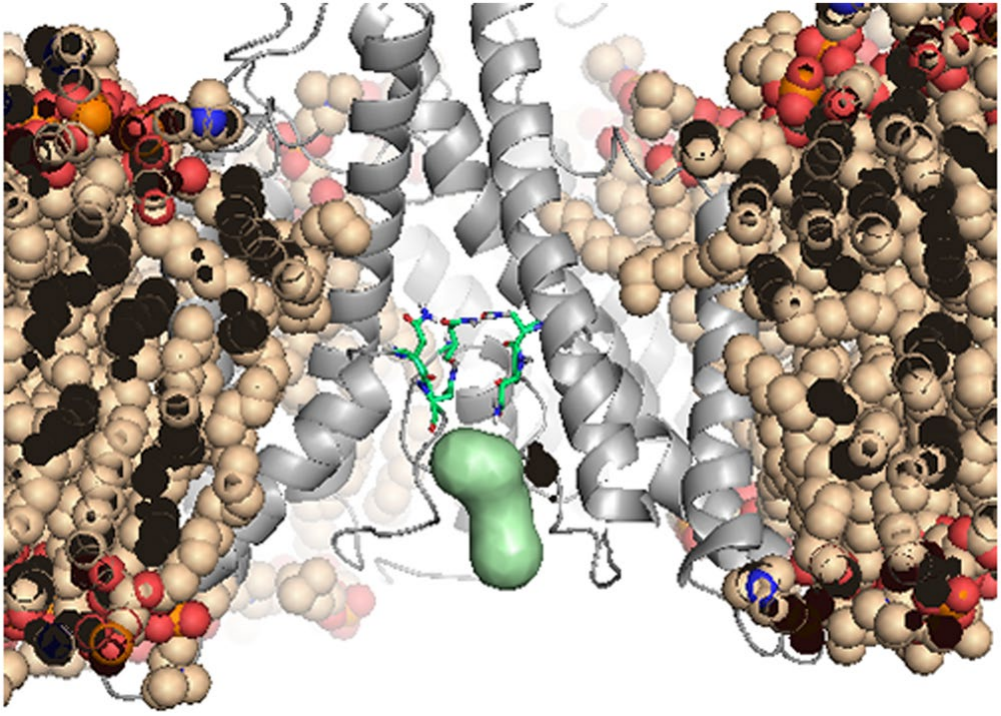
Simulated surface of an NMDAR pore. Example of the pore surface (green) inside the pore region of the NMDAR’s TMD (grey) embedded in the DOPC bilayer (wheat) under control conditions. Green sticks indicate the asparagines of the Mg^2+^ binding site (N and N + 1 site).

**Figure 7:**
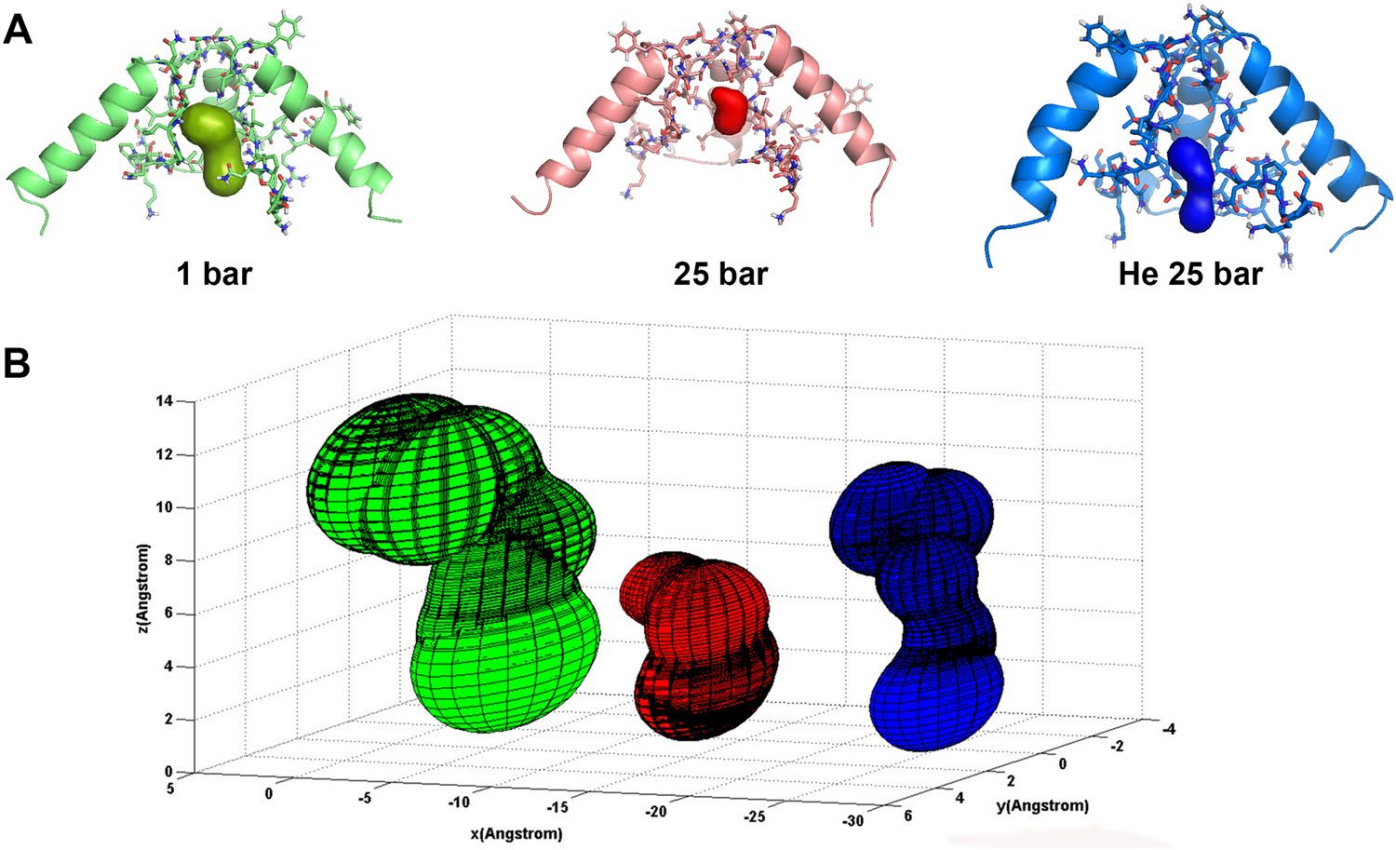
Simulated surface of an NMDAR pore under different pressure conditions. (**A**) Pore surface position in the TMD of the NMDAR under different conditions. (**B**) Simulated pore surface of the NMDAR under control conditions at 1 bar (green), at 25 bar hydrostatic pressure (red) and in helium at 25 bar (blue).

To construct the pore surface, we used Mole 2 and MatLab® software (details in Methods). The 3D model revealed that the pore surface actually bordered on the Mg^2+^ binding site (N and N + 1 sites, Fig. 6). Figure 6 presents the pore surface (green) inside the pore region of the NMDAR’s TMD (grey), embedded in the DOPC bilayer (wheat), under control conditions. Green sticks indicate the Asn of the Mg^2+^ binding site (N and N + 1 sites). Although it was expected that the pore would be different for control and high pressure simulation, due to the anticipated open or closed states, we were surprised to discover differences between the two high pressure conditions (Fig. 7).

Pore surface under hydrostatic pressure was half its size under control conditions along the Z and X axes, whereas under high pressure helium it was only slightly smaller along the Z axis and almost half its size along the X axis. In addition, pore shape would appear to be preserved under helium but not hydrostatic pressure *per se*.

When we compared the pore formation among different clusters under the same conditions (the last 50 ns of the simulation), we saw that they are very similar in shape although not identical (see examples Figs S4–5).

### Mg^2+^ binding site

**Figure 8:**
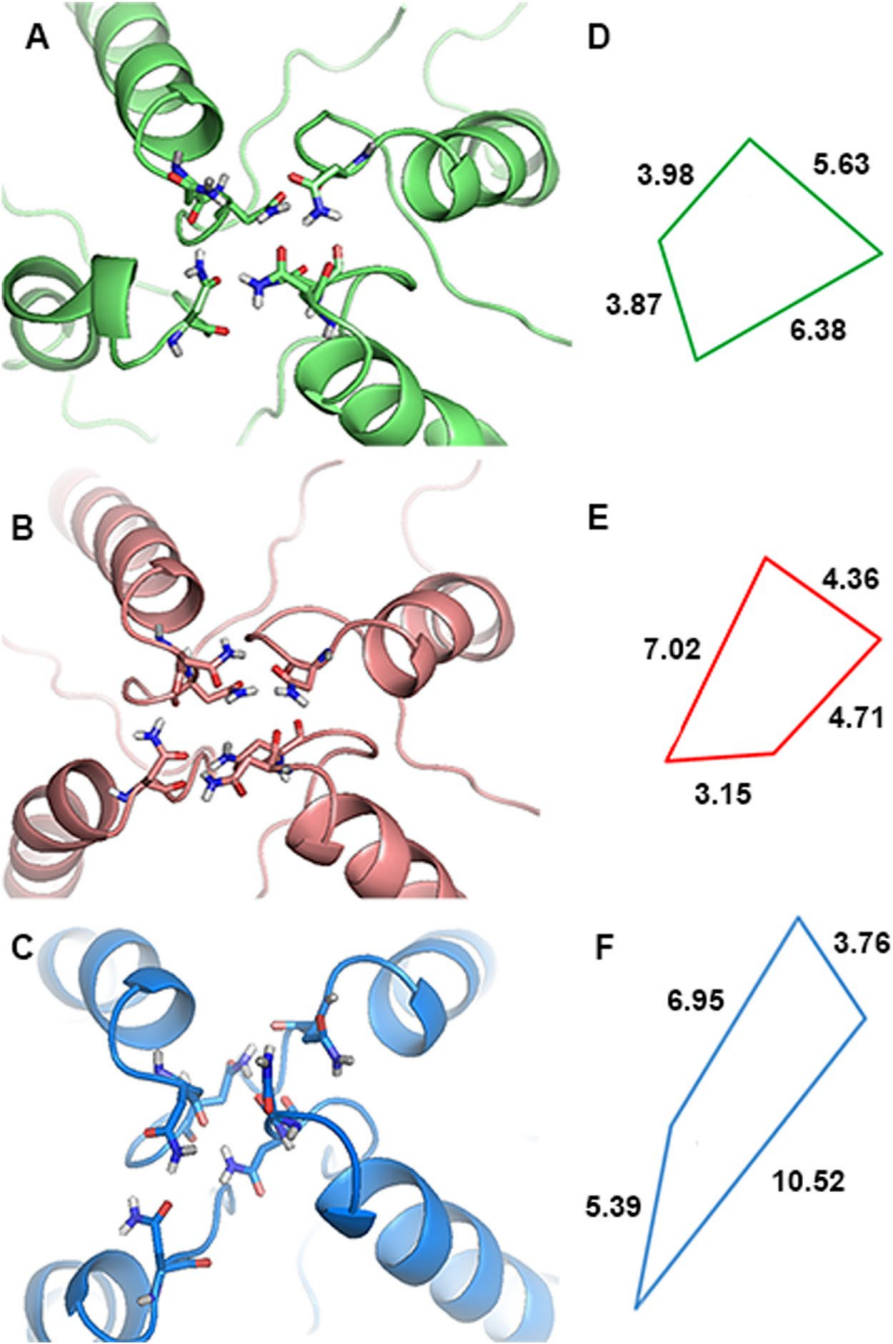
NMDAR asparagine (Asn) residue coordinations at the Mg^2+^ site. (**A**‒**C**) Asn residues shown as sticks colored by element, on the M2 region of the TMD under different simulation conditions: (**A**) Lime - 1 bar, (**B**) Salmon - 25 bar, (**C**) Marine - in helium at 25 bar. (**D**‒**F**) Projection of the OD1 atoms of the N606 (Asn of GluN1-1a, 2 subunits) and N598. (Asn of GluN2B, 2 subunits) on the X-Y plane for the three simulation conditions, with the same color coding. The distances in the X-Y plane are shown in Å.

Mor & Grossman^12^ have shown in rat brain slices that the efficacy of the Mg^2+^ block of NMDAR currents is greatly reduced under high pressure helium. MD simulations allow closer examination of the Mg^2+^ site. It contains six Asn residues in the M2 region of the TMD at the tip of the selectivity filter^35^. Zooming in on the region (Fig. 8A–C) enables measurement of the distance between four Asn residues as well as their spatial alignment (N sites only, OD1 atoms of the N606 [GluN1-1a, 2 subunits] and N598 [GluN2B, 2 subunits], Fig. 8). The rectangular-like plane (a projection on the X-Y plane of the atom coordinates) obtained for control and hydrostatic pressure (Fig. 8D,E) is converted to a diamond-like shape under high pressure helium (Fig. 8F). This result was unexpected; we had anticipated a major difference between control and high pressure, due to dissociation of the agonists from the receptor (depicting a closed state) under both high pressure conditions. Instead, the major change occurred only for high pressure helium.

### M4 α-helix analysis

**Figure 9:**
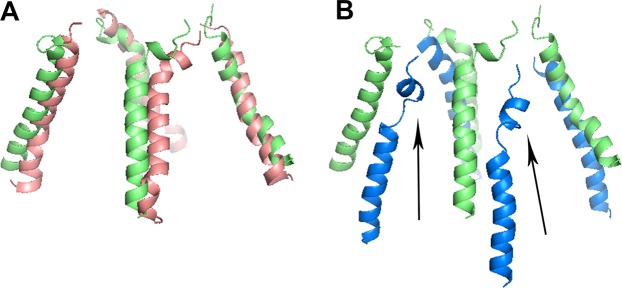
Simulated M4 α-helix (TMD) for four subunits of the NMDAR. (**A**) Alignment between control (1 bar, lime) and hydrostatic pressure (25 bar, salmon) of all four M4 α-helices in the TMD. (**B**) Similar alignment between control conditions (lime) and high pressure helium (25 bar, marine) of all four M4 segments. Note that the 3D structure under high pressure helium is quite distorted, also demonstrating helix degradation at two sites (arrows).

The M4 peripheral TMD α-helix segment, which interacts with the pore domain of the neighboring subunits, is critical for NMDAR activation and desensitization^33^. Alignment of the M4 segments from the two high pressure simulations with that of the control revealed no significant structural changes under hydrostatic pressure (Fig. 9A). However, under high pressure helium two of the α-helixes (one from the GluN1-1a and one from the GluN2B subunit) demonstrated partial degradation of the α-helix shape at the upper part of the helix (Fig. 9B). Since M4 has been identified as an important region for receptor gating^33^, it is conceivable that high pressure helium might affect receptor conductance.

## Discussion

We have used MD simulation to gain a better understanding of the molecular mechanism underlying NMDAR hyperactivity under high pressure. A total of 100 ns MD simulation of the NMDAR structure (taken from the crystal model, Uniprot entry code: 5iou), embedded in a DOPC lipid bilayer, including water and ions (Mg^2+^, Cl^−^, Na^+^, Ca^2+^ and K^+^), were performed under different pressure conditions: 1 bar, 25 bar, and in helium at 25 bar.

The RMSD values of NMDAR indicated that under control conditions (1 bar) the protein is quite stable after its relaxation from the crystal form. To our surprise, the RMSD of the protein under high pressure helium was much higher than under hydrostatic pressure alone. This probably indicates that helium induces much more distortion and instability in the NMDAR than does hydrostatic pressure *per se*. Performing cluster analysis served to strengthen this assumption, because high pressure helium clustering demonstrated much greater numbers and varieties of receptor tertiary structures than had appeared under hydrostatic pressure alone.

Slow motion viewing of the simulation and the most probable conformation of the box showed that helium causes substantial distortion of the membrane, represented as an X shape. In addition, density calculations along the Z axis showed the inflation response of the membrane to high pressure helium, as previously reported by Moskovitz & Yang for neon^30^.

Although all of the above data indicate considerable inflation and distortion of the membrane under high pressure helium, we should not forget the limitations of MD simulation. We simulate only a small patch of the membrane, and not the “closed vesicle” as in a real cell. DOPC does not include all components of the membrane, such as cholesterol. Furthermore, NMDARs usually cluster with each other and AMPAR, but our model has only one receptor. In addition, the simulation was performed on an NMDAR molecule without its C-terminal domain (CTD), an intracellular region responsible for many control and stabilization mechanisms, no membrane potential was applied, and there were artificial periodic boundary conditions. On the other hand, the effects of anesthetic and diatomic noble gases on globular proteins have been studied recently by Zhang *et al*.^41^, who found that a large bubble of dissolved gas persistently appeared under standard conditions and was adsorbed on the protein surface. Taking into account all of the above mentioned considerations, we may conclude that high pressure helium, unlike the heavy noble gases, preferentially diffuses into the hydrophobic core of the DOPC, which is consistent with helium’s inability to create complexes with macro-molecules in the bulk polar solvent^42^. Furthermore, considering the fractal order in which micro-compartments of molecular size are distributed throughout the human body, it would be logical to consider the possibility, even with respect to helium, that sufficiently high levels of local gas concentrations may be present in the central nervous system^43^. This accumulation of helium in the membrane core would cause distortion and increases in volume, even if it appeared in the form of flattened dynamic microbubbles floating at membrane cores poorly stapled by transmembrane complexes.

Noble gases pool within DOPC bilayer reaches saturation for relatively short time 10–40 ns (Moskovitz and Yang^30^, Fig. 1B), whereas a transmembrane protein slow conformational dynamics operates in the time range exceeding 100 ns. Helium bubble stability at 50–100 ns time frame of the MD trajectory in the present simulations were quantified by series of standard error calculations for helium-a.a. RDFs (Fig. 4A). These showed that only few a.a. 560–620 (chains C, D) and 780–820 (chains A, B) exhibit average RDF fluctuations exceeding 10%. NMDA RMSD plot (Fig. 2) suggests that the protein structure being exposed to hydrophobic helium phase probably starts its equilibration at 80 ns and may continue re-ordering beyond 100 ns MD time.

The detailed water solvent layouts along NMDAR subunits at 1 and 25 bar studied with RDFs demonstrate significant redistribution of channel solvation patterns, even among ‘tween’ chains of the protein subunits. Hydrophobic gating in the inner pore of a channel constituted by hydrophobic residues^37^ could be further intensified by high pressure helium, with support for this from our data for helium-dependent dehydration of the protein TMD. The water layout analysis further indicates that hydrostatic pressure *per se* does not change the water distribution within the channel in the ‘closed state’ by any structural modification (agonists are detached). It is rather the high pressure helium that succeeds in dehydrating the channel region, also preventing the accumulation of water in the vicinity of other domains of the protein; no RDF peaks were observed in the normalized layout. Our findings also indicate that phospholipid membrane receptor-protein interaction plays an important role in the high pressure response. For instance, lipids can affect functionality of the channel through channel fenestrations^44^. Helium in its turn can affect lipid-protein interaction either by reducing or enlarging channel fenestration. This further suggests that we cannot regard compression with noble gases in the same way as hydrostatic pressure alone^45^. The substantial change in –S_ch_ under high pressure helium may reinforce our presumption regarding the involvement of distorted lipid hydrophobic chains in channel modulation. It should be noted, however, that inclusion of the embedded protein in the box might reverse the trend toward alteration of –S_ch_, because in the absence of proteins in the pure lipid phase, gases tend to distribute uniformly along acyl chains and actually increase their order parameter^30^.

Simulation of both high pressure conditions revealed that the NMDAR lost its agonists, and should therefore be considered as remaining in a closed state. However, pore surface analysis indicated that there is a great difference between hydrostatic pressure and high pressure helium. The pore surface in helium demonstrated much better conservation of the tertiary structure as it had been in the “open state” than it did under hydrostatic pressure (Fig. 7). Considering the relatively stable tertiary structure of the pore region (an M2 α-helix), we may speculate that although the agonists dissociated from the ABD, the “pore” did somehow still remain in the “open state”. This may be one possible explanation for NMDAR hyperactivation in high pressure helium (see Introduction).

Furthermore, the TMD M4 α-helix, which is crucial for the gating and conductance of the receptor^33^, is altered under high pressure helium (Fig. 9). This may explain the “increased conductance” of NMDAR demonstrated in our recent study^1^. Once again, no alterations were observed in the hydrostatic pressure simulation.

Another region of the TMD, the Mg^2+^ binding site, was altered under pressure simulation. The importance of the six Asn residues of the M2 region and their role in channel permeation and Mg^2+^ blockade have been shown previously^34^. This region is also important for Ca^2+^ ion binding and its further propagation through the pore or channel^36^. The alterations observed in our simulation of high pressure helium may result in the abolition of voltage-dependent Mg^2+^ inhibition, which may explain the greatly reduced inhibition of Mg^2+^ under helium previously reported by our laboratory^12^. Conformation changes in this important region may also affect the pore’s ability to transfer ions, which may be one possible explanation for the reduction of current in NMDARs containing GluN1-1 and GluN2B subunits, as shown experimentally by Mor *et al*.^29^.

One purpose of the present study was to take a closer look at atomic level modifications in the NMDAR exposed to various high pressure conditions. Since pressure can be mediated by gases, we attempted to answer the long- speculated question: “Do noble gases at high pressure have the same effect on the protein as does hydrostatic pressure *per se*?” Because noble gases are considered to be inert, it was generally assumed they would have very little influence, if at all, on the large structures of the protein. Our analysis of the simulations suggests the opposite. However, these theoretical simulations are relatively new and require to be treated with caution, taking into consideration all of the limitations mentioned above. Nevertheless, taken together, the findings of the present study and our previous experimental results suggest, in contrast with previous models, that helium at high pressure and not hydrostatic pressure *per se* alters the receptor tertiary structure via protein-lipid interactions. This produces changes in the physiology and activity of the receptor.

HPNS symptoms are usually reversible upon decompression. However, it is interesting to note in recent reports^8^ the suggestion that repetitive exposure to high pressure over a period of years may cause chronic impairment of memory and motor function in professional divers. The pressure to which these divers may have been exposed was either just sub-threshold to HPNS or even supra-threshold, but HPNS symptoms were antagonized by the use of narcotic gas mixtures such as “trimix” containing oxygen, nitrogen and helium. As we have previously hypothesized^1^ even if no clear symptoms of HPNS are observed, the glutamate NMDAR response is still potentiated, causing more Ca^2+^ ions to flow into the neurons. An overload of Ca^2+^ may activate metabolic cascades via a number of signal transduction pathways, resulting in deterioration of the neuron and eventually leading to cell death via apoptosis^46^. These long-term health effects are therefore not a separate phenomenon, but rather an accumulation of minute deleterious changes due to the potentiation of NMDARs during each deep dive. They represent a permanent consequence of only part of the wider symptoms and signs of HPNS.

The MD simulation approach we adopted in the present study to examine the role of the NMDAR in HPNS provides an interesting point of view on HPNS and its underlying mechanisms. MD showed that hydrostatic pressure *per se* and high pressure helium have a different impact on the cell membrane and the tertiary receptor structure. Professional divers who exhibit symptoms of HPNS usually perform their dive breathing a gas mixture that contains helium. This, together with the findings of our MD simulation, enables us to speculate that it is the helium in the diver’s breathing mixture which may contribute in part to the appearance of symptoms. Further support for this theory may be found in the fact that some marine mammals are able without harm to perform deep breath hold dives, during which they are exposed only to hydrostatic pressure. The results of our simulations have shown that hydrostatic pressure *per se* has a less devastating influence on the membrane and the protein, resulting in less malfunction of the CNS.

## Materials and Methods

### Simulation box preparation

Membrane blocks (DOPC solvated in water) were extracted and replicated from the ‘control’ simulation box used in previous study^30^. 475 pre-equilibrated DOPC molecules, solvated with 104902 water molecules, were reproduced by means of a10ps-long MD fusion process for the lipids and the solvent blocks. For NMDAR construct, we used a cryo-EM structure of NMDAR containing two GluN1-1a and two GluN2B subunits with bound agonists (Uniprot entry code: 5iou)^47^. Although the resolution is only 7 Å, it was the best available choice for our study at that time because it has the whole receptor (with the exception of the CTDs) in the open state with bound Gly and Glu, and presented the smallest number of mutations. The data were refined using a 3D protein structure refinement server^48–50^. The refined NMDAR molecule was inserted into the center of an enlarged DOPC bilayer using the “gmx membed” tool^51^, resulting in final box dimensions of 14.8 × 11.9 × 20.0 nm, containing 457 lipid molecules and 103720 water molecules. The extended “simple point charge SPC” water model was used and PBC were employed. For the large NMDAR-DOPC complex, Berger and Gromos54a7 force fields were mixed and applied for describing molecular interactions^52^. Water molecules were randomly replaced by 750 Cl^−^, 50 Mg^2+^, 70 Ca^2+^, 90 K^+^, and 500 Na^+^ ions, ensuring an overall neutral electrical charge in the box. An additional 3500 water molecules were randomly replaced by atoms of helium (a noble gas) treated as simple Lennard-Jones (LJ) sites with interaction parameters ε = 0.084 kJ mol^−1^, σ = 0.256 nm^53^. The chosen number of gas atoms in the box referenced to a 3.5% molar concentration, while 2.8% was established as a threshold for dissolved anesthetic noble and diatomic gases, defining their ability to create a continuous phase (bubbles) under standard conditions within a 100 ns time range^41^.

### MD simulation

The GROMACS 5.0.4 package^54^ was used to perform MD simulations of the NMDAR embedded in the membrane at different pressures, in the presence and absence of helium. The Titan Cray XK7 supercomputer (at the Oak Ridge National Laboratory) was used for our calculations^55^. All simulation systems were energy minimized by the steepest descent method for 5*10^4^ steps followed by subsequent equilibration of NVT and NPT for 100 and 1000 ps prior to an MD production phase lasting 100 ns. The pressure range tested was 1 and 25 bar, at a constant temperature of 310 K determined at the gel-crystalline phase^56^.

The Parrinello-Rahman and Nose-Hoover methods were used to maintain the NPT ensemble, while bulk solvent and protein-bilayers were controlled separately with a relaxation time of 0.2 ps; the gas atoms were coupled to the solvent phase. Semi-isotropic pressure control was employed. A time step leapfrog integrator was used every 2 fs, and the linear constraint solver (LINCS) algorithm was applied to preserve the bond lengths. The non-bonded pair list was updated every 10 steps with a cutoff of 1.2 nm. For the short-range van der Waals interactions, a cutoff distance of 1.2 nm was used. The long-range electrostatic interactions were treated by the particle mesh Ewald method, with a grid spacing of 0.16 nm; cubic interpolation was adopted. The volume compressibility was chosen to be 4.5 × 10^−5^ (bar^−1^). Data were collected every 2 ps.

### Periodic boundary condition removal

PBC caused impaired continuity of biomolecules, which is crucial for embedded protein conformational analysis. PBC were therefore removed from all trajectory frames at the end of the simulations. We used the following set of GROMACS commands to perform this geometrical transformation:

As a first step, the “broken” bonds were connected with the biomolecules that crossed the simulation box boundary:

gmx trjconv –s md.tpr –f traj.xtc –o traj-whole.xtc –pbc whole

Second, the protein was centered in the box:

gmx trjconv –s md.tpr –f traj-whole.xtc –o traj-center.xtc –center

Finally, all atoms were placed as close as possible to the center of the box:

gmx trjconv –s md.tpr –f traj-center.xtc –o traj-compact.xtc –pbc mol –ur compact

### Clustering NMDARs

Because of differences between the NMDAR conformations for each of the simulation time points, we used the GROMACS command “gmx cluster “, with a cutoff of 0.5 nm, to cluster the different conformations from the final 50 ns of each ~100 ns simulation in groups. We used the ‘gromos’ algorithm described in Daura *et al*.^57^. At the end of the clustering process, the frame that represented the centroid of the most probable group was printed as a PDB file. All further analyses were performed on this file. It is important to note that we also analyzed cluster 2–5 in high pressure helium and hydrostatic pressure simulations. No significant changes were observed; comparison of RMS values between cluster 1 and 2, or 3, 4, 5 were similar about 1.5 Å, and therefore these results are not shown. In contrast, the RMS between control and He 25 bar and 25 bar were 9.549 Å and 8.661 Å respectively, which are considered very dramatic.

### Analyses of the NMDAR and DOPC

The following analyses were performed on the NMDAR and DOPC conformation:

RMSD calculation ‒ RMSD was calculated using the GROMACS command “gmx rms”, describing the mean deviation (nm) of NMDAR atoms along the time axis from the initial NMDAR structure at 0 ns^58^.

Density along the Z axis ‒ mass density (kg/m3) of the gas, DOPC and NMDAR along the Z axis was calculated using the GROMACS command “gmx density”. This performed density histogram binning relative to the center of an arbitrary group, in absolute box coordinates^59^.

Radial Distribution Functions (RDF) ‒ RDF for gas and SPC solvent (water) were calculated using the GROMACS command ‘gmx rdf’ for index files containing every 5th Cα sequence from each NMDAR chain as a reference particle. A similar approach was employed for testing noble gas distribution along DOPC molecules in a pure bilayer^29^. Calculations were performed automatically using a self-developed bash script, at 50–100 ns for each 5 ns sampling interval separated by a 10 ns skipping interval to ensure a statistically independent data ensemble. Statistical analysis of the resulting functions was carried out using a local C# script.

Volume calculation – we calculated the volume of a structure (nm3) for a given probe size by rolling a virtual probe on the surface of the macromolecule. The calculations were performed for DOPC, NMDAR and helium confined between two DOPC monolayers, using a probe size identical to a water molecule of 3.0 Å^60^.

Order parameter calculation ‒ the order parameter for chains Sn-1 and Sn-2 of a DOPC molecule was calculated using a new analytical tool which enables rapid and accurate calculation of -S_ch_ for a united atom lipid^39^. The method explicitly considers *prochirality* of acyl chains, the descriptors pro-R and pro-S serving to distinguish between two identical substituents attached to a hybridized carbon atom.

Pore structure ‒ Mole 2 software^61^ was employed to represent pore surface; the data collected were later used for visualization of the pore with MatLab® software.

Images of all molecular structures and their alignment were created using PyMol software^62^.

## Supplementary information

Supplementary figures

## References

[1] Bliznyuk A, Aviner B, Golan H, Hollmann M, Grossman Y (2015). The N-methyl-D-aspartate receptor’s neglected subunit - GluN1 matters under normal and hyperbaric conditions. The European journal of neuroscience.

[2] Bliznyuk A, Gradwohl G, Hollmann M, Grossman Y (2016). The Enigma of the Dichotomic Pressure Response of GluN1-4a/b Splice Variants of NMDA. Receptor: Experimental and Statistical Analyses. Frontiers in molecular neuroscience.

[3] Bliznyuk, A., Golan, H. & Grossman, Y. Marine Mammals’ NMDA Receptor Structure: Possible Adaptation to High Pressure Environment. *Frontiers in Physiology* **9**, 10.3389/fphys.2018.01633 (2018). 10.3389/fphys.2018.01633 PMC6262034 30524300

[4] Ciesielski, T. & Imbert, J.-P. In *Offshore Technology Conference*. (Houston, Texas, 1989).

[5] Lafay V, Barthelemy P, Comet B, Frances Y, Jammes Y (1995). ECG changes during the experimental human dive HYDRA 10 (71 atm/7,200 kPa). Undersea & hyperbaric medicine: journal of the Undersea and Hyperbaric Medical Society, Inc.

[6] Bennett, P. B. & Rostain, C. In *Bennett and Elliott’s Physiology and Medicine of* Diving (eds Brubakk, A. O. & Neuman, T. S.) 323–357 (Saunders, 2003).

[7] Grossman, Y., Aviner, B. & Mor, A. In *Comparative High Pressure Biology* (ed P. Sébert) 161–186 (Taylor & Francis 2010).

[8] Gronning M, Aarli JA (2011). Neurological effects of deep diving. Journal of the neurological sciences.

[9] Daniels, S. & Grossman, Y. In *Bennett and Elliott’s Physiology and Medicine of* Diving (eds Brubakk, A.O., T.S. Neuman, A. O. & Elliott, D. H.) 265–299 (Saunders, 2003).

[10] Fagni L, Soumireu-Mourat B, Carlier E, Hugon M (1985). A study of spontaneous and evoked activity in the rat hippocampus under helium-oxygen high pressure. Electroencephalography and clinical neurophysiology.

[11] Fagni L, Zinebi F, Hugon M (1987). Helium pressure potentiates the N-methyl-D-aspartate- and D,L-homocysteate-induced decreases of field potentials in the rat hippocampal slice preparation. Neuroscience letters.

[12] Mor A, Grossman Y (2010). The efficacy of physiological and pharmacological N-methyl-D-aspartate receptor block is greatly reduced under hyperbaric conditions. Neuroscience.

[13] Zinebi F, Fagni L, Hugon M (1988). Decrease of recurrent and feed-forward inhibitions under high pressure of helium in rat hippocampal slices. European journal of pharmacology.

[14] Zinebi F, Fagni L, Hugon M (1990). Excitatory and inhibitory amino-acidergic determinants of the pressure-induced neuronal hyperexcitability in rat hippocampal slices. Undersea biomedical research.

[15] Traynelis SF (2010). Glutamate receptor ion channels: structure, regulation, and function. Pharmacological reviews.

[16] Dingledine R, Borges K, Bowie D, Traynelis SF (1999). The glutamate receptor ion channels. Pharmacological reviews.

[17] Furukawa H, Singh SK, Mancusso R, Gouaux E (2005). Subunit arrangement and function in NMDA receptors. Nature.

[18] Paoletti P (2011). Molecular basis of NMDA receptor functional diversity. The European journal of neuroscience.

[19] Watanabe M, Inoue Y, Sakimura K, Mishina M (1992). Developmental changes in distribution of NMDA receptor channel subunit mRNAs. Neuroreport.

[20] Akazawa C, Shigemoto R, Bessho Y, Nakanishi S, Mizuno N (1994). Differential expression of five N-methyl-D-aspartate receptor subunit mRNAs in the cerebellum of developing and adult rats. The Journal of comparative neurology.

[21] Laurie DJ, Seeburg PH (1994). Regional and developmental heterogeneity in splicing of the rat brain NMDAR1 mRNA. *The*. Journal of neuroscience: the official journal of the Society for Neuroscience.

[22] Monyer H, Burnashev N, Laurie DJ, Sakmann B, Seeburg PH (1994). Developmental and regional expression in the rat brain and functional properties of four NMDA receptors. Neuron.

[23] Sheng M, Cummings J, Roldan LA, Jan YN, Jan LY (1994). Changing subunit composition of heteromeric NMDA receptors during development of rat cortex. Nature.

[24] Takai H, Katayama K, Uetsuka K, Nakayama H, Doi K (2003). Distribution of N-methyl-D-aspartate receptors (NMDARs) in the developing rat brain. Experimental and molecular pathology.

[25] Paoletti P, Bellone C, Zhou Q (2013). NMDA receptor subunit diversity: impact on receptor properties, synaptic plasticity and disease. Nature reviews. Neuroscience.

[26] Sanz-Clemente A, Nicoll RA, Roche KW (2013). Diversity in NMDA receptor composition: many regulators, many consequences. The Neuroscientist: a review journal bringing neurobiology, neurology and psychiatry.

[27] Mor A, Grossman Y (2006). Modulation of isolated N-methyl-d-aspartate receptor response under hyperbaric conditions. The European journal of neuroscience.

[28] Mor A, Grossman Y (2007). High pressure modulation of NMDA receptor dependent excitability. The European journal of neuroscience.

[29] Mor A (2012). Pressure-selective modulation of NMDA receptor subtypes may reflect 3D structural differences. Frontiers in cellular neuroscience.

[30] Moskovitz Y, Yang H (2015). Modelling of noble anaesthetic gases and high hydrostatic pressure effects in lipid bilayers. Soft Matter.

[31] Casado M, Ascher P (1998). Opposite modulation of NMDA receptors by lysophospholipids and arachidonic acid: common features with mechanosensitivity. The Journal of physiology.

[32] Korinek M (2015). Cholesterol modulates open probability and desensitization of NMDA receptors. The Journal of physiology.

[33] Amin JB (2017). Divergent roles of a peripheral transmembrane segment in AMPA and NMDA receptors. The Journal of general physiology.

[34] Tu YC, Kuo CC (2015). The differential contribution of GluN1 and GluN2 to the gating operation of the NMDA receptor channel. Pflugers Arch.

[35] Wollmuth LP, Kuner T, Sakmann B (1998). Adjacent asparagines in the NR2-subunit of the NMDA receptor channel control the voltage-dependent block by extracellular Mg2. The Journal of physiology.

[36] Mesbahi-Vasey S, Veras L, Yonkunas M, Johnson JW, Kurnikova MG (2017). All atom NMDA receptor transmembrane domain model development and simulations in lipid bilayers and water. PloS one.

[37] Aryal P, Sansom MSP, Tucker SJ (2015). Hydrophobic Gating in Ion Channels. Journal of Molecular Biology.

[38] Engel AK, Cowburn D (1981). The origin of multiple quadrupole couplings in the deuterium NMR spectra of the 2 chain of 1,2 dipalmitoyl-sn-glycero-3-phosphorylcholine. FEBS Letters.

[39] Piggot TJ, Allison JR, Sessions RB, Essex JW (2017). On the Calculation of Acyl Chain Order Parameters from Lipid Simulations. Journal of Chemical Theory and Computation.

[40] Tajima N (2016). Activation of NMDA receptors and the mechanism of inhibition by ifenprodil. Nature.

[41] Zhang L (2017). Inert Gas Deactivates Protein Activity by Aggregation. Scientific Reports.

[42] Weathersby PK, Homer LD (1980). Solubility of inert gases in biological fluids and tissues: a review. Undersea biomedical research.

[43] Doolette DJ, Upton RN, Grant C (2005). Perfusion–diffusion compartmental models describe cerebral helium kinetics at high and low cerebral blood flows in sheep. The Journal of physiology.

[44] Kaczmarski JA, Corry B (2014). Investigating the size and dynamics of voltage-gated sodium channel fenestrations. Channels.

[45] Dodson BA, Furmaniuk ZW, Miller KW (1985). The physiological effects of hydrostatic pressure are not equivalent to those of helium pressure on Rana pipiens. The Journal of physiology.

[46] Orrenius S, Zhivotovsky B, Nicotera P (2003). Regulation of cell death: the calcium-apoptosis link. Nature reviews. Molecular cell biology.

[47] Zhu S (2016). Mechanism of NMDA Receptor Inhibition and Activation. Cell.

[48] Bhattacharya D, Cheng J (2013). i3Drefine software for protein 3D structure refinement and its assessment in CASP10. PloS one.

[49] Bhattacharya D, Cheng J (2013). 3Drefine: consistent protein structure refinement by optimizing hydrogen bonding network and atomic-level energy minimization. Proteins.

[50] Bhattacharya D, Nowotny J, Cao R, Cheng J (2016). 3Drefine: an interactive web server for efficient protein structure refinement. Nucleic acids research.

[51] Wolf MG, Hoefling M, Aponte-Santamaria C, Grubmuller H, Groenhof G (2010). g_membed: Efficient insertion of a membrane protein into an equilibrated lipid bilayer with minimal perturbation. Journal of computational chemistry.

[52] Berger O, Edholm O, Jahnig F (1997). Molecular dynamics simulations of a fluid bilayer of dipalmitoylphosphatidylcholine at full hydration, constant pressure, and constant temperature. Biophysical journal.

[53] Verlet L, Weis J-J (1972). Perturbation theory for the thermodynamic properties of simple liquids. Molecular Physics.

[54] Hess B, Kutzner C, van der Spoel D, Lindahl E (2008). GROMACS 4: Algorithms for Highly Efficient, Load-Balanced, and Scalable Molecular Simulation. J Chem Theory Comput.

[55] Abraham MJ (2015). GROMACS: High performance molecular simulations through multi-level parallelism from laptops to supercomputers. SoftwareX.

[56] Booker RD, Sum AK (2013). Biophysical changes induced by xenon on phospholipid bilayers. Biochimica et biophysica acta.

[57] Daura, X. *et al*. Peptide Folding: When Simulation MeetsExperiment. *Angew. Chem. Int. Ed*., 249–253 (1999).

[58] Maiorov Vladimir N., Crippen Gordon M. (1995). Size-independent comparison of protein three-dimensional structures. Proteins: Structure, Function, and Genetics.

[59] *gmx density*, http://manual.gromacs.org/archive/5.0.3/programs/gmx-density.html.

[60] Voss NR, Gerstein M (2010). 3V: cavity, channel and cleft volume calculator and extractor. Nucleic acids research.

[61] Sehnal D (2013). MOLE 2.0: advanced approach for analysis of biomacromolecular channels. Journal of cheminformatics.

[62] The PyMOL Molecular Graphics System v. 1.8 (LLC, 2015).

