# Supplementary material for: The effect of high pressure on the NMDA receptor: molecular dynamics simulations
Source: Sci Rep. 2019 Jul 25;9:10814. doi: 10.1038/s41598-019-47102-x (PMC6658662; doi:10.1038/s41598-019-47102-x)
Supplement: Supplementary file 1 — Supplementary figures [file 41598_2019_47102_MOESM1_ESM.pdf]

The effect of high pressure on the NMDA receptor: molecular dynamics simulations

Alice Bliznyuk[1,2*], Yoram Grossman[2] & Yevgeny Moskovitz[3]

[1]Israel Naval Medical Institute, Haifa, Israel. [2]Department of Physiology and Cell Biology, Faculty of Health Sciences, and Zlotowski Center for Neuroscience, Ben-Gurion University of the Negev, Beer-Sheva, Israel. [3]Institute of Natural Sciences and Mathematics, Ural Federal University, Yekaterinburg, Russia.

*Corresponding author

email: shuturov@gmail.com (A.B.)

Supplementary figures

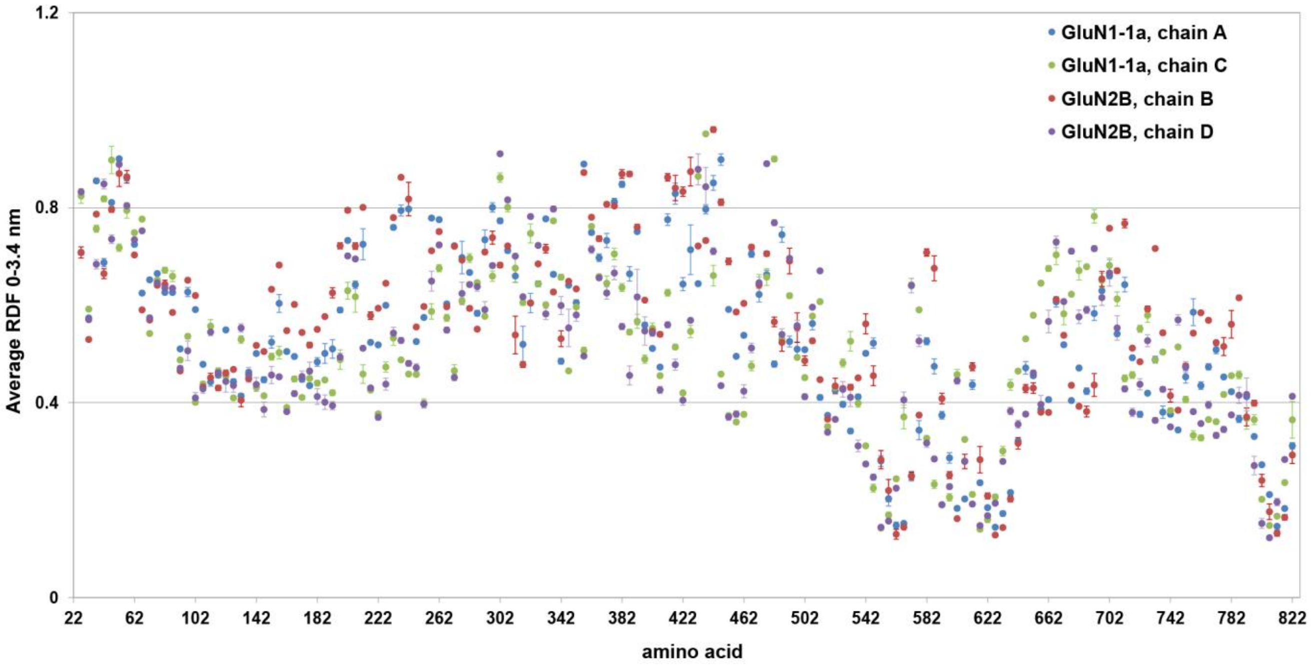

Figure S1. Average values for SPC solvent (water) radial distribution function (RDF) around Cα along A, B, C and D chains of the NMDAR molecule in the range 0–3.4 nm under control conditions (1 bar).

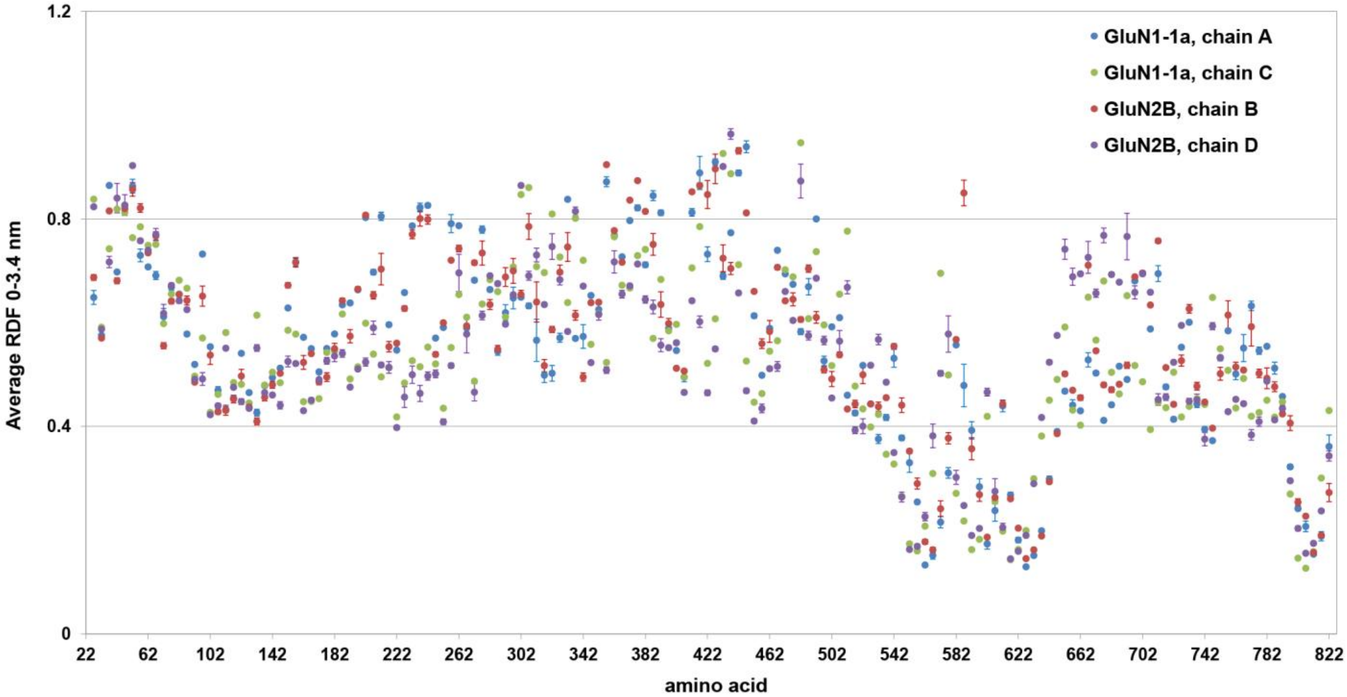

Figure S2. Values for water RDF around Cα along A, B, C and D chains of the NMDAR molecule in the range 0–3.4 nm at 25 bar hydrostatic pressure.

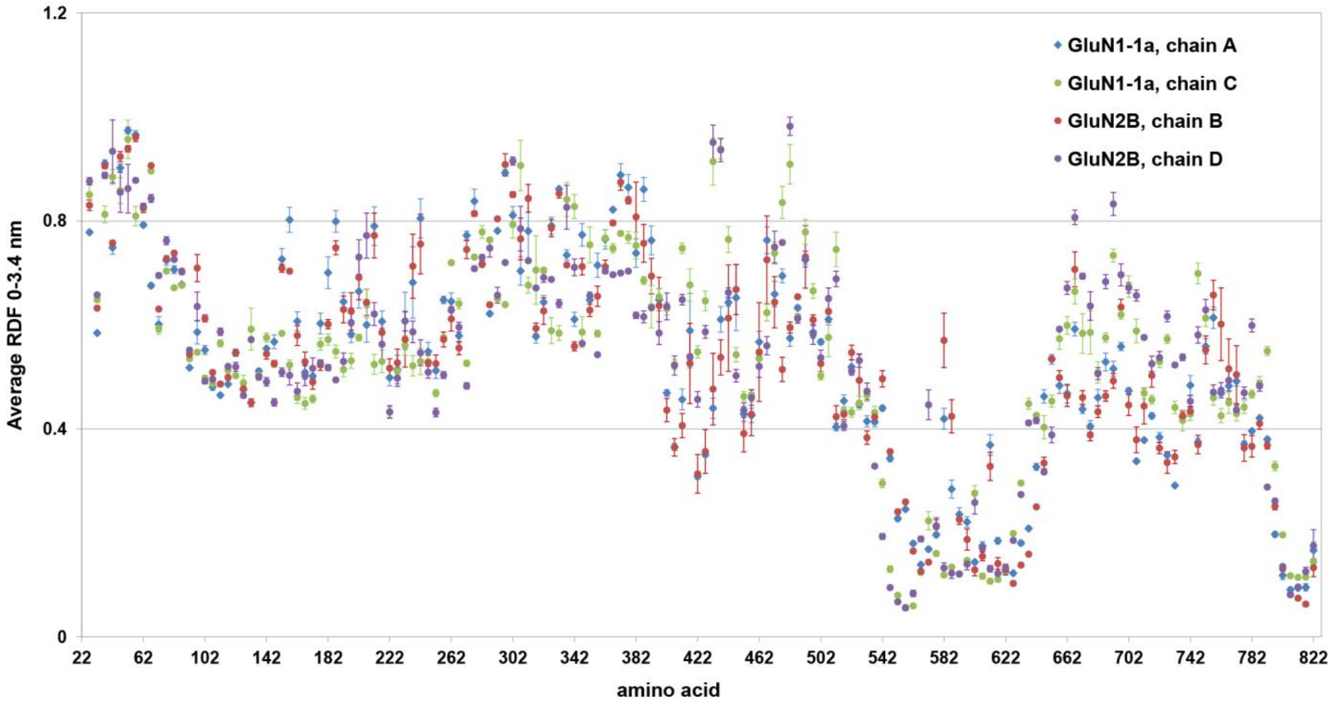

Figure S3. Values for water RDF around Cα along A, B, C and D chains of the NMDAR molecule in the range 0–3.4nm in helium at 25 bar.

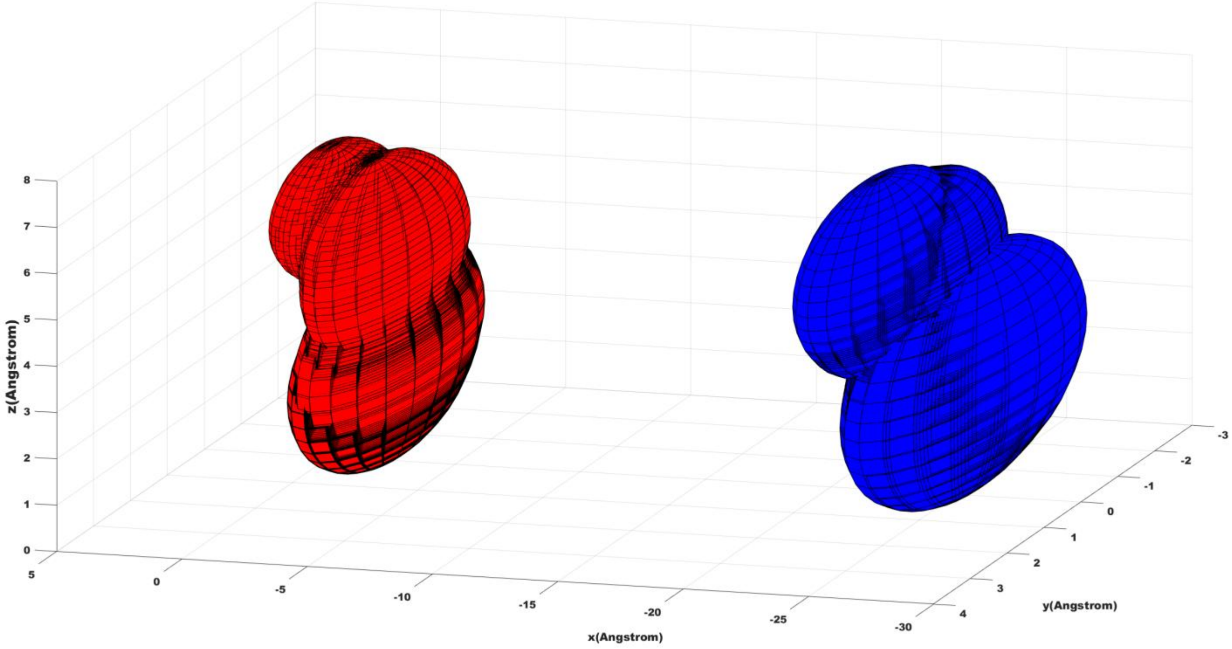

Figure S4. Pore surface 25 bar, cluster 1-red, cluster 3-blue.

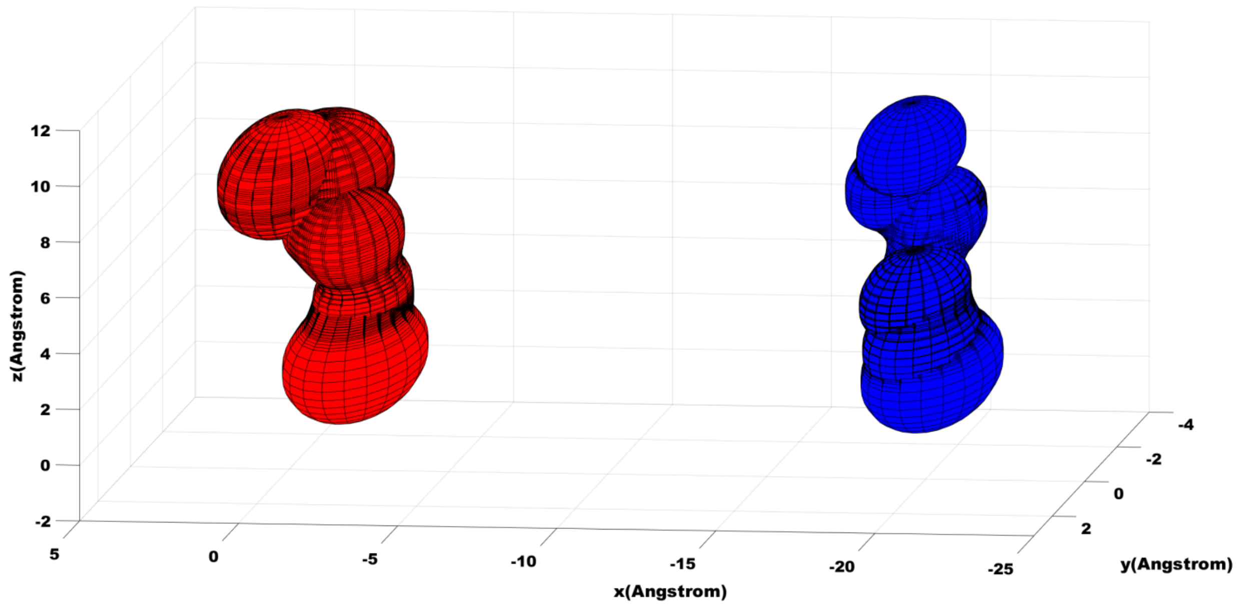

Figure S5. Pore surface He 25 bar, cluster 1-red, cluster 3-blue.
